# Accuracy Assessment of EM3D App-Based 3D Facial Scanning Compared to Cone Beam Computed Tomography

**DOI:** 10.3390/dj12110342

**Published:** 2024-10-25

**Authors:** Selene Barone, Alessandro Antonelli, Marianna Salviati, Vincenzo Greco, Francesco Bennardo, Kathrin Becker, Amerigo Giudice, Michele Simeone

**Affiliations:** 1 Department of Health Sciences, School of Dentistry, Magna Graecia University of Catanzaro, Viale Europa, 88100 Catanzaro, Italy; selene.barone@unicz.it (S.B.); alessandro.antonelli@unicz.it (A.A.); marianna.salviati@studenti.unicz.it (M.S.); vincenzo.greco001@studenti.unicz.it (V.G.); a.giudice@unicz.it (A.G.); 2 Department of Orthodontics and Dentofacial Orthopaedics, Charité-Universitätsmedizin Berlin, Aßmannshauser Str. 4-6, 14197 Berlin, Germany; kathrin.becker@charite.de; 3 Department of Neurosciences, Reproductive Sciences and Dentistry, School of Dentistry, University of Naples “Federico II”, Via Sergio Pansini, 14, 80131 Napoli, Italy; michele.simeone@unina.it

**Keywords:** 3D facial imaging, smartphone, TrueDepth scanner, stereophotogrammetry, CBCT

## Abstract

**Background:** The use of 3D facial scans is becoming essential for dental practice. However, traditional scanners require labor-intensive procedures and are expensive, making them less accessible in routine clinical practice. In this context, high-performance smartphones and dedicated apps offer a more accessible alternative. This study aims to validate the accuracy of the EM3D app, which utilizes the iPhone’s TrueDepth camera technology, by comparing it to Cone Beam Computed Tomography (CBCT). **Methods:** Thirty patients requiring CBCT scans were recruited for the study. Facial scans obtained with the TrueDepth camera of the iPhone 13 Pro in conjunction with EM3D app were automatically superimposed onto the 3D models derived from the CBCTs through the implementation of a deep learning methodology. The approach enabled the automatic identification of fifteen landmarks to perform linear and angular measurements for quantitative assessment. A color map was created to highlight discrepancies between the overlaid meshes, and the overall surface differences between the models were automatically quantified. **Results:** The overall surface difference between the CBCT and EM3D scans was highly accurate, with a mean discrepancy of 0.387 ± 0.361 mm. The mean discrepancies of most measurements were lower than 1 mm (five out of six; 83.33%) between the groups, with no significant differences (*p* > 0.05). **Conclusions:** The combination of the iPhone’s TrueDepth camera and the EM3D app exhibited high accuracy for 3D facial modeling. This makes it a cost-effective alternative to professional scanning systems.

## 1. Introduction

The facial scanning procedure employs advanced three-dimensional acquisition technologies, such as 3D scanners, stereoscopic cameras, or photogrammetry, to capture specific anthropometric details of an individual’s face and create an accurate three-dimensional model [1]. Facial scans generate data that can be used for various analyses, including patient diagnosis, treatment planning, and communication between providers, patients, and laboratory technicians, to achieve more predictable results [2]. Additionally, integrating extraoral tissue and facial structure data with intraoral scans and Cone Beam Computed Tomography (CBCT) allows for creating a virtual patient, improving precision in anatomical detection [3].

In orthodontics, the digital workflow plays a pivotal role, combining CBCT scans with extraoral facial optical data and/or intraoral optical data to diagnose dentofacial deformities, assess the interaction between hard and soft tissues, monitor and evaluate changes over time, and plan orthognathic surgery in the most severe cases. The precision in capturing facial details is essential for assessing dental position, bone structure, and facial relationships, especially in cases of orthognathic surgery that require detailed planning to guarantee high accuracy in the visualization of soft-tissue profile prediction, allowing daily acquisition in clinical practice without additional radiation exposure for the patient [4,5].

In prosthodontics, the use of a virtual patient enhances the planning and execution of the prosthetic-implant project. Facial scanning information is incorporated into a CAD/CAM workflow, expediting the rehabilitation procedure and leading to more predictable, functional, and aesthetic outcomes [6,7].

Facial scanning, in conjunction with other advanced imaging technologies, has recently emerged as a key player in enhancing the precision of oral surgical procedures and aiding in the analysis of post-operative outcomes, such as the assessment of facial swelling [8]. Additionally, 3D facial scans have also been used to predict severe clinical conditions, such as the presence of Obstructive Sleep Apnea (OSA), where the relationship between craniofacial anomalies and the upper airway is assessed through a mathematical algorithm involving linear and geodesic craniofacial measurements from 3D photography [9].

Several methods for capturing facial scans exist and various systems have been developed over the years. Even without a specific facial scanner, the reconstruction of soft tissues can be obtained using a patient’s CBCT.

This is accomplished through the segmentation process, wherein the density of voxels selected within the entire dataset results in a volumetric surface reconstruction. It is critical to note that CBCT is an invasive procedure, exposing patients to ionizing radiation. Therefore, using CBCT exclusively to scan facial soft tissue is not justified for patient safety [10]. The leading technologies used to detect facial surfaces without employing ionizing radiations are photogrammetry, stereo photometry, structured light scanning, and laser scanning [2]. These methods have been widely documented and utilized to evaluate different endpoints: photogrammetry involves capturing multiple photographs from various angles to generate a detailed, precise 3D model of the face; scanners using the structured light acquisition method also provide a viable option, demonstrating remarkable levels of accuracy and precision [11,12,13,14]. Regardless of the scanning method employed, each offers significant advantages due to their non-invasiveness, accuracy, and reproducibility [15]. However, it is essential to consider that such devices may require more laborious scanning procedures and, notably, that they are associated with significant expenses and prohibitively high costs.

For this reason, they are not readily available in standard dental practices. In this context, high-performance and widely available devices like smartphones offer a more accessible alternative. When paired with specialized applications, smartphones enable rapid, safe, cost-effective, and non-invasive facial scans, providing sufficiently accurate results for dental use [16]. The EM3D application (EM3D App by Ethan Makes, https://em3dscanningapp.com—accessed on 22 March 2022), available in the Apple Store, uses the TrueDepth camera technology built into recent iPhone models (starting with the iPhone X) to generate three-dimensional facial reconstructions. The TrueDepth camera uses a dot projector to project over 30,000 dots onto the surface in front of the device; the infrared camera then captures the reflection of these dots and uses the resulting pattern to build a surface model. To ensure that this detection remains accurate in low-light conditions, the system includes an infrared illuminator for the area in front of the camera [17]. Other applications using similar technologies have been developed and validated in previous studies, such as Bellus3D Face Camera Pro (Bellus3D Inc., Campbell, CA, USA) [16], ScandyPro App (ScandyPro, New Orleans, LA, USA) [18], Capture (Standard Cyborg Inc., San Francisco, CA, USA) [19], and Face2Gene (FDNA Inc., Boston, MA, USA) [20]. To the contrary, the EM3D application has yet to be analyzed in previously published research. Furthermore, unlike other applications such as Bellus3D, which is no longer available in the Apple Store, EM3D remains accessible, making it a viable alternative.

This study aims to validate the EM3D application by evaluating its accuracy in comparison with CBCT, the gold-standard method for capturing detailed anatomical features.

## 2. Materials and Methods

### 2.1. Study Design

This study was designed as a prospective cohort study in a single-center cross-sectional investigation. It was conducted in accordance with the Declaration of Helsinki and approved by the Regional Ethics Committee (n. 465/2020).

### 2.2. Study Sample and Data Collection Method

The study sample consisted of 30 healthy volunteer patients, requiring CBCT scans with a large Field of View (FOV; 17 × 20 cm) for different clinical purposes. The sample size was determined based on a previous study, which calculated that a minimum of 21 subjects would be sufficient to achieve a statistical power of 0.80 with an α level of 0.05 [18]. All participants were fully informed about the study procedures and the processing of their personal data. Written informed consent was obtained from each patient.

All CBCT scans were acquired with the same device (Viso^®^ G7, Planmeca, Helsinki, Finland) and stored in a dedicated database. Immediately afterward, facial scans were acquired using the TrueDepth camera of an iPhone 13 Pro (Apple Inc., Cupertino, CA, USA) in conjunction with the EM3D application. All 3D facial scans were carried out by the same dentist and the scanning procedure was standardized: patients maintained a stationary head position, facing forward, with an intercuspal position, closed mouth, and relaxed lips. Simultaneously, the operator rotated the device around the patient’s head to complete the scan registration. The scanning environment remained constant for all patients, and artificial lights were employed to control and ensure identical lighting conditions for each acquisition. For the CBCT scan, the patient’s head was aligned to ensure that the Frankfurt plane was parallel to the floor and head supports were attached to ensure the patient’s stability during acquisition. All the facial scans were exported in Standard Triangle Language (STL) format, and all CBCT data were exported in Digital Imaging and Communications in Medicine (DICOM) format.

### 2.3. Data Management and Analyses

**Figure 1:**
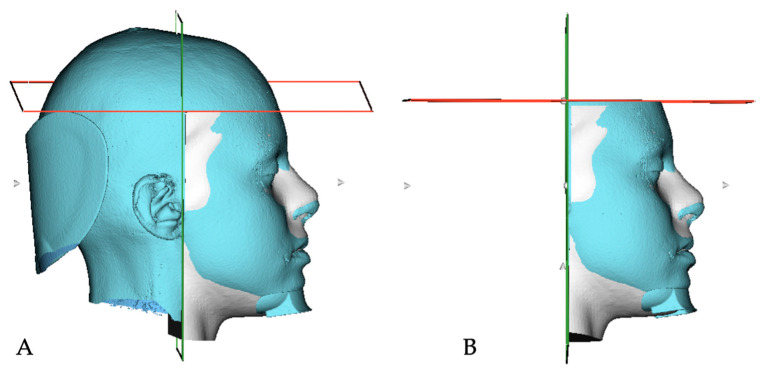
(**A**) Automatic registration of the scan on the model obtained from the CBCT and (**B**) definition of the anatomical limits of the scan.

Following data anonymization using a pseudonym system to preserve each patient’s privacy, the operator used the 3DSlicer software (Version 5.6.2, 2024) on a Windows computer (Intel(R) Xeon(R) w5-3425, 64GB RAM, Nvidia RTX A5000). While this machine was utilized for optimal efficiency, all procedures can also be conducted on standard laptops with moderate performance [21]. An automated and standardized orientation of the CBCT scans was performed using the Automatic Standard Orientation (ASO) tool, according to specific reference planes: the Frankfurt Horizontal (FH) plane and the mid-sagittal plane [22]. Subsequently, automatic segmentations of soft tissues from the CBCT scans were generated using the Automated Dental Tool [23]. The operator exported the segmented models in the Visualization Toolkit (VTK) file format. Facial scans acquired thought EM3D were imported in 3D Slicer using the Standard Triangulation Language (STL) format. The scans acquired using the application were automatically registered and superimposed onto the 3D models segmented from CBCT, which is considered the gold standard, through the SlicerCMF plug-in. Anatomical parts not involved in the facial analysis, such as the neck, ears, and hair, were removed to enhance the accuracy of the superimposition process (Figure 1).

**Figure 2:**
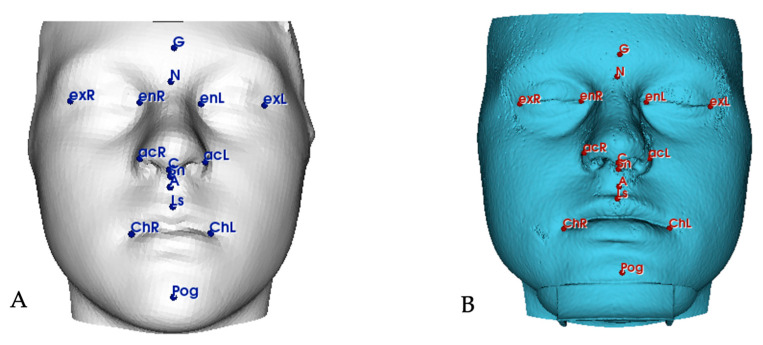
Automatic identification of 15 skin reference landmarks for automatic calculation of linear and angular measurements. (**A**) Scan performed with EM3D; (**B**) 3D model obtained from CBCT.

Fifteen anatomical skin points (Table 1), representing key reference landmarks for morphological analysis, were selected on the models (Figure 2) [24]. For quantitative assessment, the Tools of Automated Landmark Identification and Quantification were used to automatically detect points and calculate specific linear and angular measurements (Table 2) [19,25,26,27]. The qualitative evaluation highlighted the visualization of eventual discrepancies between the two superimposed meshes (soft tissue segmentation and facial scan), creating a colormap with a specific color bar where green indicates the absence of variations, red indicates outward displacement of the facial scan, and blue indicates inward displacement. Finally, using the MeshStatistics tool, the overall surface discrepancies between the models were automatically quantified.

### 2.4. Statistical Analysis

Sample data were collected using Microsoft Excel (Version 16.0, Microsoft Corporation, 2023). Statistical data analysis was performed using the statistical software program RStudio (Version 1.4.1717, RStudio Team, 2021). The Shapiro–Wilk test was used to assess the normality of the data distribution. For normally distributed variables, the mean and standard deviation (SD) were reported along with a 95% confidence interval (CI). For asymmetric variables, median and interquartile range (IQR, Q3-Q1) were used to present the descriptive statistics. To highlight any significant difference between the CBCT and EM3D measurements, the bivariate statistical analysis included the Student’s *t*-test for normal variables and the Wilcoxon test for asymmetric distributions.

## 3. Results

**Figure 3:**
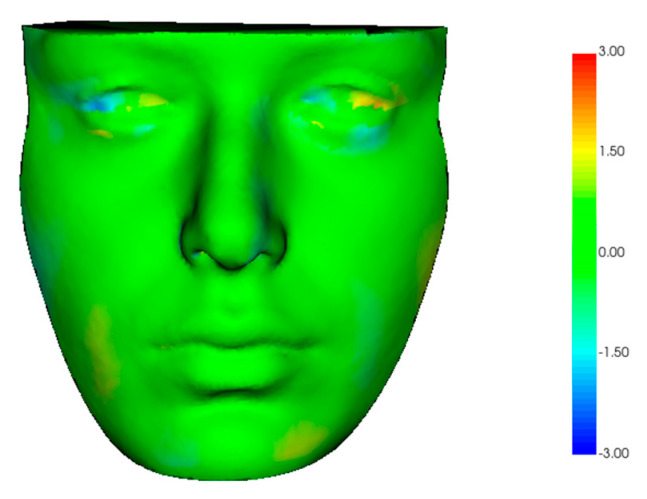
Qualitative analysis using colormap obtained by superimposition of EM3D scan on the model obtained from CBCT. Green indicates the absence of variations, red indicates outward displacement of facial scan, and blue indicates inward displacement. The colormap showed high accuracy in facial scanning with low discrepancy in the eye region.

The final study sample included 30 patients (17 F and 13 M) with a mean age of 27.6 ± 6.8 years. The qualitative analysis showed a high degree of overlap between the two scans, where the absence of variation was expressed by green models (Figure 3).

Quantitative analysis recorded linear, angular, and surface discrepancies; descriptive statistics are reported in Table 2. The bivariate analysis did not highlight any statistically significant difference between the measurements conducted on the models obtained from CBCT and EM3D scan (*p* > 0.05).

**Figure 4:**
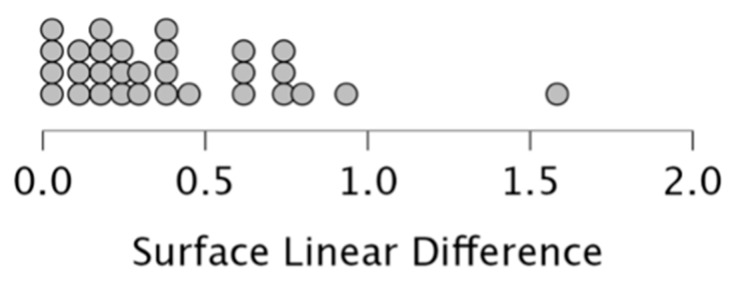
Dot-plot showing the distribution of the overall surface discrepancy between CBCT segmentations and EM3D scans.

The overall surface difference reported high values of accuracy (mean discrepancy: 0.387 ± 0.361 mm). Considering the entire sample, 21 demonstrated a high degree of accuracy with a surface difference lower than 0.5 mm, eight were accurate (range: 0.5–1 mm), and only one exceeded 1 mm (Figure 4).

Regarding the linear measurements, the mean values exhibited high accuracy, with discrepancies between the models mostly lower than 1 mm (Table 2).

Angular measurements recorded higher differences compared to linear measurements, but no significant discrepancies were reported between the two models (*p* > 0.05) (Table 2).

## 4. Discussion

This study aimed to evaluate the accuracy of facial scans recorded using the iPhone’s TrueDepth camera in conjunction with the EM3D application. Scans were compared with 3D models of the patient’s face obtained from their respective CBCT scans to assess their accuracy. According to Ghamri et al., the segmentations derived from CBCT scans were used as the reference method due to their high accuracy, estimated to be around 0.12 mm with a standard deviation of 0.5 mm [10].

Although several studies have investigated different scanning apps and compared them to professional scanners with satisfactory results, some of these apps have yet to be made available [19,28]. Moreover, no studies have specifically analyzed the EM3D application, mainly reporting the overall surface accuracy of the registered scans. This study differentiates itself by comparing an iPhone scanning app (EM3D) with a medically validated method, specifically Cone Beam Computed Tomography (CBCT), which allows for detailed acquisition of anatomical structures. The measurements were automatically calculated using a dedicated tool, which allows a direct comparison between the entire surface of the meshes obtained from EM3D and CBCT and has the great advantage of avoiding any landmark localization or distance calculation made by the operator.

This analysis showed a mean surface linear difference between the two models of 0.387mm ± 0.361mm, which is deemed highly reliable [29]. A recent study conducted by Andrews et al. on the Bellus3D application in comparison with the 3dMDface system (3dMD Inc., Atlanta, GA) revealed a mean overall trueness indicated by a mean Root Mean Square (RMS) of 0.86 ± 0.31 mm for the Bellus3D Face App. They found the highest accuracy for the Nasion and Pronasale points and less reliability for points near the mouth or nose, such as the Subnasale point [16]. These findings were confirmed by the results of this study, showing that the C-$\hat{Sn}$-Ls angle is the least accurately measured. This angle is defined by three specific landmarks: the columella, subnasal point, and labial superius. All of them are located in the concave region between the base of the nose and the upper lip, which is more obscured due to its anatomical characteristics [16]. This suggests that acquiring scans using the iPhone’s TrueDepth camera should be performed cautiously because it may be less accurate in more curved and hidden areas, such as the subnasal region. The same conclusion was defined by D’Ettore et al., who compared two iPhone applications, Bellus3D and Capture, with the 3dMDface scanner. Their findings indicated that the colormaps obtained from the 3D surface comparison suggest that the flat parts of the face, such as the forehead, chin, and cheeks, are the most precise. In contrast, the least accurate areas were those rich in curvature (mouth, lips, and eyes) [19]. Furthermore, the colormaps in this study showed a reduction in accuracy within the ocular region, as was also pointed out in the study by Thurzo et al. They superimposed scans from the Bellus3D application with segmentations derived from CBCT using colormaps, highlighting that the deeper structures, especially the orbital region, showed lower accuracy, with variations up to 3 mm, which is considered unacceptable [20]. Although these areas did not show the highest accuracy level in this analysis, no regions with surface differences greater than 3 mm were observed. This discrepancy may be due to the different methodologies used for model superimposition and difference calculation, which are fully automated in this workflow compared to their semi-automated approach. Furthermore, the lower accuracy in recording the orbital zone is probably attributable to the methodology employed in acquiring the scan, which utilizes the iPhone’s TrueDepth camera in conjunction with the associated application. The acquisition process is relatively lengthy, and during this time, even if the patient maintains a steady forward gaze, they tend to blink repeatedly, which can affect the accuracy of the scan.

Alternative methods for analyzing facial scanning systems have been documented in the literature, including comparisons with direct anthropometric measurements. Piedra-Cascón et al. evaluated the accuracy of the Bellus3D app, reporting an absolute mean of 0.91 ± 0.32 mm, calculated from the linear discrepancies with direct anthropometry [15]. Although this result is promising, it may be influenced by direct anthropometric assessments, in which minor fluctuations in posture or facial features are unavoidable and can introduce errors and artifacts. This study addressed this limitation by utilizing models segmented from CBCT as a reference, which is regarded as the gold standard. Liu et al. also compared the Bellus3D application and the 3dMDface scanner with direct anthropometric measurements on a mannequin head. The results demonstrated that both scanning methods exhibited considerably higher accuracy than previously reported in other studies. However, using a mannequin head in their research instead of human participants makes these results less reliable [28]. It is crucial to underscore that most studies utilizing the iPhone’s TrueDepth camera have employed the Bellus3D application. Despite the extensive corroboration of its reliability, Bellus3D is no longer accessible on the App Store. The available analysis application, EM3D, prompted this investigation, which offers a viable substitute for this technology. According to Rudy et al., an important aspect is that the TrueDepth scanner integrated into Apple smartphones and tablets offers significant advantages in terms of cost and accessibility, which effectively lowers the barrier to entry for three-dimensional technology [18].

Scans can be completed quickly with real-time processing and, crucially, they can be conducted without exposing the patient to radiation. This technology is not only precious for private dental practices, where it offers a practical alternative to purchasing professional scanners that can be very expensive, but also for telemedicine, as patients are able to conduct the scans independently and transmit the data to their clinicians for remote assessment, providing a reliable and efficient means of patient care. Moreover, the strength of this study lies in the comparison between two different methods within the same patient, minimizing the variability seen across different patient populations. This approach ensures that the findings are applicable to diverse ethnic groups outside the European context, as the focus was on intra-patient assessments rather than inter-patient differences.

This study’s major strength is the use of a fully automated 3D workflow of imaging analysis. This methodology, based on a deep-learning approach, was used to identify and locate the landmarks needed to evaluate the linear and angular measurements and, more importantly, for the automated superimposition of the models and for the calculation of the overall surface discrepancy between them. This approach helped to minimize the risk of human error. It should be noted that this study is subject to several limitations. Firstly, the small sample size might impact the generalizability of the findings. As CBCT with a large FOV was selected as the reference method, only patients with a clinical indication for the examination were included in the study; this was a necessary step to justify the exposure to ionizing radiation for pathological reasons. Another limitation concerns the use of chin support during the CBCT acquisition. Although the patients received detailed indications to stay relaxed during the exam, this may result in artifacts in the lower portion of the face where the skin is in contact with the support.

## 5. Conclusions

This study confirmed the accuracy of 3D facial models generated using the EM3D app combined with the iPhone’s TrueDepth camera by comparing them with models derived from CBCT scans, which is considered the gold standard. The EM3D app demonstrated high accuracy for flat areas of the face, such as the forehead and chin, and a lower but acceptable precision in more curved and complex areas, such as the subnasal and orbital region. This analysis confirmed the potential use of the EM3D app, representing a viable alternative to expensive professional scanning systems. Smartphone-based technologies provide a more accessible and potentially accurate option for facial documentation, clinical planning in dentistry, and post-operative dental monitoring.

Further studies will be supported to expand the study sample and generalize the results on a global scale to ensure and confirm the applicability of the results obtained.

## Figures and Tables

**Table 1:** Landmarks Definition.

Name	Abbreviation	Definition
Glabella	G	Most prominent midpoint between the eyebrows
Nasion	N	Deepest point of the nasal bridge
Exocanthion *	Ex (R or L)	Outer commissure of the eye fissure
Endocanthion *	En (R or L)	Inner commissure of the eye fissure
Alare *	Ac (R or L)	Most lateral point on nasal alar contour
Columella	C	Most anterior point of the nasal septum
Subnasale	Sn	Midpoint of the base of the nose
A point	A	Deepest point of the philtrum
Labiale superius	Ls	Midpoint of the upper vermillion line
Cheilion *	Ch (R or L)	Most lateral point of the labial commissure
Pogonion	Pog	Most prominent point of the chin on the sagittal midline

* Bilateral Landmarks.

**Table 2:** Bivariate analysis of linear and angular measurements of CBCT segmentation model and EM3D model.

	Landmarks	CBCT		EM3D		Difference		
		Mean Value	SD	Mean Value	SD	Mean Value	SD	*p* Value
Linear Measurements (mm)	ExR-ExL	91.869	5.09	91.845	4.65	0.023	2.50	0.986
	EnR-EnL	33.751	3.03	33.355	3.17	0.395	1.34	0.642
	ChR-ChL	48.978	3.52	48.155	5.10	0.822	2.89	0.494
	AcR-AcL	33.486	3.89	33.564	3.90	−0.078	1.29	0.941
	G-Sn	60.977	5.07	61.323	4.41	−0.346	1.94	0.790
	Sn-Pog	48.431	6.81	49.722	5.94	−1.291	2.92	0.461
Angle Measurements (°)	N-$\hat{Sn}$-Pog	162.667	9.85	161.137	8.44	1.529	3.72	0.543
	C-$\hat{Sn}$-Ls	117.479	17.06	121.048	16.40	−3.569	7.54	0.437

## Data Availability

The data presented in this study are available on request from the corresponding author.
